# Multi-type child maltreatment: prevalence and its relationship with self-esteem among secondary school students in Tanzania

**DOI:** 10.1186/s40359-018-0244-1

**Published:** 2018-07-24

**Authors:** Adela A. Mwakanyamale, Dickson P. Wande, Yu Yizhen

**Affiliations:** 1 Department of Maternal and Child Health, School of Public Health, Tongji Medical College, Huazhong University of Science and Technology, Wuhan, 430030 Hubei China; 2 School of Pharmacy, Muhimbili University of Health and Allied Sciences, United Nations road, Dar es salaam, Tanzania

**Keywords:** Multi-type child maltreatment, Self-esteem, Secondary school students, Tanzania

## Abstract

**Background:**

Child maltreatment is becoming predominantly multi-type in nature. Studies report that multi-type child maltreatment is associated with low self-esteem in adolescence and adulthood. There is a lack of published studies in Tanzania regarding multi-type child maltreatment and its relationship with self-esteem in adolescence. This study investigates the prevalence of multi-type child maltreatment and its relationship with self-esteem among secondary school students in Tanzania.

**Methods:**

A cross-sectional, community-based study of secondary school students was conducted in randomly selected secondary schools in Tanzania. A multistage cluster sampling technique was employed to obtain the required number of study participants. The Rosenberg Self-Esteem Scale and the Adverse Childhood Experiences (ACE) questionnaire were used to measure the variables under investigation in the study. A total of 1000 participants (M: F ratio = 1.2:1) were studied. The mean age at presentation was 16.24 ± 7.36 years. The modal age group was 16–18 years (54.2%).

**Results:**

The prevalence of multi-type child maltreatment was 97.6%. The prevalence of physical abuse, physical neglect, emotional neglect emotional abuse and sexual abuse was 82.1, 26.2, 51.9, 21.8 and 24.7%, respectively. Females reported a higher prevalence of physical abuse (84.3%), physical neglect (28.0%) and sexual abuse (26.2%) than their male counterparts. Emotional abuse (53.3%) was reported more often by males. In terms of ACE, participants were classified as having zero (2.4%), one (22.4%), two (20.3%), three (18.2%), four (14.7%), five (12.8%) and over five (9.2%) types of maltreatment.

With regard to multi-type child maltreatment, emotional abuse (X^2^ = 2.925, *p* = 0.001), emotional neglect (X^2^ = 2.329, *p* = 0.032), physical neglect (X^2^ = 22.508, *p* < 0.001) and physical abuse (X^2^ = 6.722, *p* = 0.036) were significantly associated with low self-esteem.

**Conclusion:**

The current study demonstrates that multi-type child maltreatment exists in Tanzania and has adversely affected self-esteem among secondary school students. We believe that this study has significantly added to the body of literature on child maltreatment by investigating exposure to 10 types of ACEs as opposed to single types, as the majority of previous studies have investigated.

## Background

Maltreatment in children is a worldwide health problem; if interventions are not well executed, maltreatment can have lifelong impacts on victims [1]. Child maltreatment is any physical or emotional mistreatment, sexual abuse or neglect that harms the child’s health, survival, dignity or development [2]. Maltreatment can be classified into five different forms of abuse (e.g., emotional, sexual or physical) and two different forms of neglect (emotional or physical) [3].

Studies report that the global prevalence of child maltreatment varies significantly depending on how one defines child maltreatment, the measurements s/he employs, the characteristics of the sample involved as well as the methodologies involved. The prevalence ranges from 5 to 83% for each form of child maltreatment [4]. The World Health Organization (WHO) estimated that there are 40 million children worldwide aged 0 to 14 years who are currently suffering from maltreatment, and they need urgent health and social care. Various meta-analyses have reported prevalence of 17.7% for physical abuse, 26.7% for psychological abuse, 11.8% for sexual abuse and 16.3% for neglect [5]. However, reliable global estimates for the prevalence of child maltreatment are still missing, as data for many countries, especially resource-limited countries, are lacking, and the available data represent only a small percentage of the magnitude of the problem. Generally, Africa has the highest prevalence rate of all forms of child maltreatment, whereas Asia has the lowest rates of sexual abuse [6].

The focus of studies on the prevalence and effects of child maltreatment have constantly been changing in the past few years [7]. In the past, each type of maltreatment was frequently investigated individually, as reflected in many clinical and community-based studies. It is now particularly clear that no form of child maltreatment occurs alone, as has been thought and assessed in many studies in the past several years [8].

No child experiences only one form of maltreatment. Most often, children experience multiple forms of maltreatment. As a result, the health outcomes they experience will also be an interplay of multiple forms of maltreatment, in accordance with the different combinations of maltreatment experienced [9]. In many different countries, community-based studies have shown that many young people reported of having experienced more than one type of maltreatment during childhood [10]. Multi-type maltreatment is a simultaneous process whereby a child or a baby experiences more than one or varied forms of maltreatments [11]. As highlighted earlier in this paper, multi-type maltreatment subsumes neglect or abuse (physical, sexual, and emotional) and family violence. The extent of a child’s exposure to multiple forms of maltreatment in developing countries such as Tanzania is a conundrum, as it is not well studied and requires sensitive analysis. [12].

Several studies have revealed that children’s experiences of multi-type maltreatment have mental and physical health consequences similar to those of children with exposure to single-type maltreatment. In fact, exposure to varied forms of maltreatment has tangible physical health consequences, and it negatively affects mental health status [13]. The affected mental health may result in depression, low self-esteem and/or anxiety disorders. It also augments the risk of attempts to commit suicide and drug abuse and may lead to long-term negative effects on academic performance and employability [14]. Generally, experiencing physical and/or emotional abuse in childhood results in deviant sexual behaviour, low self-esteem, difficulties in dealing with psychosocial challenges and anger during adulthood [15].

Notwithstanding the fact that much is known about the existence of various types of childhood maltreatments and their consequences on children’s mental, psychological, emotional and physical development, there are currently few studies in developing countries, including Tanzania, that have examined the prevalence of multiple forms of child maltreatment and the consequences of these varied forms of maltreatment. This study sought to determine the prevalence of multiple forms of child maltreatment and to assess its relationship with self-esteem among secondary students (i.e., Form I-IV or grades 8–11) in Tanzania. Therefore, the current study specifically aimed to investigate the links among more than one form of child maltreatment and self-esteem among secondary students in Tanzania.

## Methods

### Study design

A cross-sectional study of secondary school students was conducted using a random selection of secondary schools in Tanzania. A multistage cluster sampling technique was employed to obtain the required number of the study participants.

### Participants

The target population comprised male and female students aged 13–24 years. A total of 1000 students were recruited (553 males and 447 females). As there were no previous studies in Tanzania regarding the prevalence of multi-type child maltreatment and its relationship with self-esteem, we could not calculate the sample size based on prevalence. Consequently, a convenience sample size of 1000 students (including both genders) was selected for the study.

### Measures

Two questionnaires per student were used to measure the different variables under study. The Adverse Childhood Experiences (ACE) questionnaire and the Rosenberg Self-Esteem Scale were the variables in the study.

### Adverse childhood experiences

The Adverse Childhood Experiences [16] questionnaire consisted of 38 items that assessed exposure to 10 types of ACE, including abuse. The items were adapted from the Childhood Trauma Questionnaire (CTQ) [17]. The CTQ was developed by Bernstein and Fink [17]. Through the CTQ, the participants were to rate the frequency of abuse and neglect events that occurred during their childhood or when they were growing up. The rating scale was from 0-*never true* to 5-*very often true*. The CTQ was a 70-item retrospective questionnaire, and the participants were to rate on frequencies. Sometimes, the scale can be shortened, and the shortened CTQ assesses emotional neglect, physical neglect, physical abuse, sexual abuse or emotional abuse and can have 28 items, depending on exploratory and confirmatory factory analyses.

The CTQ is suitable for adults and adolescents aged 12 and over. Additionally, the CTQ is a self-report inventory providing screens of histories of childhood abuse and neglect that are brief, reliable and valid [17]. Participants responded to a number of statements about childhood events, which are arranged according to their frequency on a 5-point Likert scale. It usually did not take more than 10 min to complete the questionnaire. CTQ items assessed exposure to ten types of ACE, including exposure to neglect (i.e., physical and emotional), abuse (i.e., emotional, physical and sexual) and household challenges (i.e., household mental illness, household substance abuse, household physical violence, parental separation/divorce, and incarcerated family members) prior to age 18.

Psychometrically, the CTQ is appropriate in community samples with the best test-retest reliability [17], displaying convergent and discriminant validity [18]. Test-retest reliabilities ranging from 0.79 to 0.86 and internal consistency reliabilities ranging from 0.66 to 0.92 have been displayed by the CTQ [19].

#### Rosenberg self-esteem scale (RSE)

This scale was developed by Rosenberg [20], and it consists of 10 self-report items that show one’s general belief about herself/himself. Each item had responses on a 4-point Likert scale, from *strongly agree* (3) to *strongly disagree* (0). Five items were reverse-scored, from *strongly disagree* (3) *to strongly agree* (0). The scale was validated on a large sample of high school students. Test-retest correlations are typically in the range of 0.82 to 0.88, and Cronbach’s alpha for various samples range from 0.77 to 0.88. The Rosenberg Self-Esteem Scale displays a test-retest reliability of 0.85. Validity scores of the RSE ranged from 0.56 to 0.67 when the results were correlated with other tests and interviewers’ ratings of self-esteem [21].

### Sampling technique

We employed multistage cluster sampling technique. First, we randomly selected 5 different regions (similar to provinces in other countries) from the mainland of Tanzania; second, we used simple random sampling in selecting 10 secondary schools in different geographical locations of the regions, and from each school, 20 students were randomly selected through simple random sampling.

### Study variables

There are two significant variables in this study: the independent variable is multiple forms of childhood maltreatment (ACE), while the dependent variable is self-esteem (Rosenberg scale).

### Statistical analysis

The Statistical Package for Social Sciences software (SPSS for windows 15.0, SPSS Inc., Chicago, IL, USA) was used in computing statistical analyses. Categorical variables were summarized through calculating proportions and frequency tables, while for continuous variables, means, standard deviations and ranges were used in summarizing the information.

Additionally, the significance of the association between the independent variable, multi-type childhood maltreatment, and the dependent variable, self-esteem, was tested using chi-square (X^2^) tests, and *p* < 0.05 was considered the level of significance. Additionally, multivariate logistic regression models yielded adjusted odds ratios (ORs) and 95% confidence intervals (CIs), which estimated the associations between self-esteem and each of the ten categories of ACE. Analytically, the numbers of ACE were summed for each respondent (ACE score range: 0–10). Later, analyses were conducted with the summed scores (1, 2, 3, 4, or < 5) as dichotomous variables (yes/no), with 0 experiences as the referent.

## Results

### Prevalence of single and multiple forms of child maltreatment

A total of 1000 participants were studied during the period of study. Out of these participants, 553 (55.3%) were males, and 447 (44.7%) were females. The ratio of males to females was 1.2:1. The ages of participants at presentation ranged from 15 to 24 years, with a mean of 16.45 ± 6.42 years. The modal age group was 16–18 years; this age group accounted for 542 (54.2%) cases.

Out of the 1000 participants, 97.6% reported experiencing more than one form of maltreatment, and 9.2% reported only one (single) form of maltreatment (ACE score).

The prevalence of physical abuse, emotional neglect, physical neglect, sexual abuse and emotional abuse was 82.1, 51.9, 26.2, 24.7 and 21.8%, respectively. Females reported a higher prevalence of physical abuse (84.3%), physical neglect (28.0%) and sexual abuse (26.2%) than males. Emotional abuse (53.3%) was reported more often by males.

In terms of ACE, participants were classified as having zero (2.4%), one (22.4%), two (20.3%), three (18.2%), four (14.7%), five (12.8%) and over five (9.2%) types of maltreatment (Table 1).

**Table 1:** Prevalence of ACE categories and ACE scores by gender

Categories of ace	Prevalence %		
	Male *n* = 553	Female *n* = 447	Total *N* = 1000
Abuse			
Sexual	23.5	26.2	24.7
Physical	80.3	84.3	82.1
Emotional	21.3	22.4	21.8
Neglect			
Emotional	53.3	50.1	51.9
Physical	24.8	28.0	26.2
Ace score			
0	3.3	1.3	2.4
1	23.3	21.3	22.4
2	21.3	19.0	20.3
3	18.4	17.9	18.2
4	13.4	16.4	14.7
5	11.6	14.3	12.8
> 5	8.7	9.8	9.2

### Exposure to household dysfunction and abuse

Several important variables under the category of household dysfunction were also analysed. Violence or a threat of violence towards a child, in the form of spanking, slapping, kicking or pushing by their parents or guardians, was the most common form of abuse experienced by the majority of student participants (76.9%) (Table 2).

**Table 2:** Frequency distribution of household dysfunction and abuse items

Household dysfunction and abuse item (Age below 18 years)	Sex		Total (N = 1000)
	Male (*n* = 553)	Females (*n* = 477)	
Death of a parent/guardian	73 (57.9)	53 (42.1)	126 (15.1)
Separated/divorced parents	85 (54.1)	72 (45.9)	157 (18.8)
Household substance abuse	47 (54.0)	40 (46.0)	87 (10.4)
Household mental illness	72 (63.7)	41 (36.3)	113 (13.5)
Criminal household members	80 (55.6)	64 (44.4)	144 (17.2)
Saw or heard a parent or household member in the home being yelled at, screamed at, sworn at, insulted or humiliated	328 (58.3)	235 (41.7)	563 (67.3)
Saw or heard a parent or household member in the home being slapped, kicked, punched or beaten up	344 (58.5)	244 (41.5)	588 (70.3)
Saw or heard a parent or household member in the home being hit or cut with an object (stick, bottle, club, knife, whip)	195 (57.4)	145 (42.6)	340 (40.7)
If a parent, guardian or other household member threatened to or actually did abandon you or throw you out of the house	119 (61.0)	76 (39.0)	195 (23.3)
If a parent, guardian or other household member yelled or screamed at you or insulted or humiliated you	334 (57.7)	245 (42.3)	579 (69.3)
If a parent or other household member spanked, slapped, kicked, punched or beat you	380 (59.1)	263 (40.9)	643 (76.9)
If a parent, guardian or other household member hit or cut you with an object (stick, bottle, club, knife, whip, etc.)	126 (57.5)	93 (42.5)	219 (26.2)

### Exposure to community violence

In this study, 80% of participants witnessed someone being physically abused. The most common occurrences of community violence reported by participants have been compiled in Table 3.

**Table 3:** Frequency distribution of exposure to community violence items

Community violence (age below 18 years)	Sex		Total *N* = 1000
	Male *N* = 553	Female *N* = 447	
Exposed to bullying	183 (56.0)	144 (44.0)	327 (39.1)
Witnessed someone being physically abused	407 (60.8)	262 (39.2)	669 (80.0)
Witnessed a threat with a weapon (knife or gun)	159 (58.7)	112 (41.3)	271 (32.4)
Forced to go and live in another place	52 (59.1)	36 (40.9)	88 (10.5)
Exposure to physical violence by authority figures (e.g., soldiers, police or militia) and/or gangs	10 (26.3)	28 (73.7)	38 (4.5)
Witnessed physical violence against a family member or friend by authority figures (e.g., soldiers, police or militia) and/or gangs	40 (54.1)	34 (45.9)	74 (8.9)

### Relationship between multiple forms of child maltreatment and scores on the Rosenberg self-esteem scale

As shown in Table 4 below, emotional abuse (X^2^ = 2.925, *p* = 0.001), emotional neglect (X^2^ = 2.329, *p* = 0.032), physical neglect (X^2^ = 22.508, *p* < 0.001) and physical abuse (X^2^ = 6.722, *p* = 0.036) were significantly associated with low self-esteem. Household dysfunction and sexual abuse were not significantly associated with low self-esteem (*p* > 0.005).

**Table 4:** Relationship between multiple forms of child maltreatment (ACE) and scores on the Rosenberg Self-Esteem Scale

Forms of ACE (dependent variable)		Rosenberg Self-esteem Scale				
	Total	Low	Normal	High	χ^2^	*P*-Value
	*N* = 1000	*N* = 17.9%	*N* = 65.8%	*N* = 16.3%		
Emotional Abuse	218	46 (21.1)	52 (23.9)	120 (55.0)	4.925*	0.001
Sexual Abuse	247	35 (14.2)	50 (20.2)	162 (65.6)	0.439	0.383
Emotional Neglect	519	82 (15.8)	101 (19.5)	336 (64.7)	2.329*	0.032
Physical Neglect	262	36 (13.7)	70 (26.7)	156 (59.5)	22.508**	< 0.001
Physical Abuse	821	136 (16.6)	135 (16.4)	550 (67.0)	6.722*	0.036
Household Mental Illness	142	28 (19.7)	23 (16.2)	91 (64.1)	1.677	0.466
Household Substance Abuse	114	20 (17.5)	22 (19.3)	72 (63.2)	0.554	0.819
Household Physical Violence	799	126 (15.8)	143 (17.9)	530 (66.3)	0.791	0.651
Parental Separation	201	36 (17.9)	42 (20.9)	123 (61.2)	4.593	0.290
Criminal Household Member	176	35 (19.9)	26 (14.8)	115 (65.3)	3.115	0.239

*P-value< 0.05

**P-value< 0.01

## Discussion

The results from this study provide insights on multi- type child maltreatment and its relationship with self-esteem among secondary school students (13–24 years old) in Tanzania. To the best of our knowledge, this is the first study in Tanzania investigating the existence of more than one type of child maltreatment and their relationships with self-esteem among secondary school students. In this study, the prevalence of multi-type child maltreatment is 97.6%; this figure is higher than 67.2 and 52.0%, which were reported by Burke et al. [22] and Felitti et al [23], respectively. Our study has revealed a low prevalence of more than one/many types of maltreatment in childhood.

This finding is lower than what McGee et al. found. They reported that 98.5% of participants had experienced more than one or varied types of maltreatment in childhood [16]. Other scholars have found even lower prevalence rates than what has been shown in the present study. On the one hand, Higgins and McCabe [24] found that 43% of their participants had experienced multi-type maltreatment during their childhood. On the other hand, Sesar et al*.* [25] found that 58% of participants had experienced more than one type of maltreatment during childhood. The reason for these differences may be partly due to methodological differences as well as different sampling demographics. The differences in the maltreatment cases used in these studies may be among the potential factors contributing to the differences in this finding.

In this study, it has been shown that physical abuse is the most commonly experienced type of maltreatment among the multiple forms that children typically experience. This finding is at odds with that of Feng et al. [26], who reported that children are most commonly exposed to violence as a type of child maltreatment. Using the child abuse screening tool (ICAST), Al-Eissa et al [27] found that the frequency of emotional abuse was high compared with other forms of child maltreatment.

Generally, the gender distribution in many studies shows a higher prevalence of physical abuse in males than in females, which reflects findings that suggest that males are more commonly victims of physical violence [8, 28]. In the study by Donget et al [29], it was reported that during childhood, females were found to experience less physical abuse than males. This finding is contrary to that of our study, in which the rate of physical abuse was found to be higher in females than in males. In Tanzanian and African culture in general, females are considered weaker than males and are hence easier to victimise.

Child neglect is a result of the failure of an individual to fulfil his/her obligation to the child, particularly in mental, physical or psychological care [28]. Neglect subsumes the greatest number of forms of child maltreatment but has been ignored and overlooked by society [30]. In our study, emotional neglect was the second-most frequent type experienced by student participants during childhood, accounting for more than half of participants (51.9%). Regarding the gender distribution, this study demonstrated that male participants were more often emotionally neglected than their female counterparts. Physical neglect has been reported in previous studies as the most common type of neglect, which can jeopardize children’s development, slow progress in body weight, lead to malnutrition and illnesses and increase the potential for physical injuries [31]. Our study found that more than 26.2% of participants reported being physically neglected, with a higher prevalence in females than in males (28.0% versus 24.8%). The prevalence of physical neglect in our study is higher than that reported in other studies [32]. We propose that the reason for this observation may be due to differences in socioeconomic status between the study settings. There is a high association between physical neglect and poor socioeconomic status [33], a situation that is widespread in Tanzania.

According to the World Health Organization (WHO), 20% of girls and 5–10% of boys had experienced sexual abuse [2]. As reported by previous authors [34], female student participants in this study also reported a significantly higher prevalence of childhood sexual abuse than their male counterparts. There is a low but significant gender difference in the prevalence of sexual abuse in developing countries (where males’ experiences of sexual abuse are higher) than in developed countries [35].

While males constantly report less sexual abuse than females, differences in prevalence and gender predomination between countries can be explained by differences in research methodologies. The reason for the higher prevalence of childhood sexual abuse among female participants in this study may be males are ashamed to report sexual abuse.

In this study, emotional abuse was the least frequent type of abuse experienced by respondents during childhood, accounting for less than 22% of participants (21.8%). Here, emotional abuse was reported more often by males than by females. In fact, children who are exposed to emotional abuse are uniquely affected. The consequences of emotional abuse can be just as severe and long-lasting, even if no physical pain or sexual contact is inflicted on a child [36]. It is frequently difficult to detach the effects of varied forms of maltreatment because the co-occurrence rate of emotional abuse and other types of maltreatment, such as physical abuse and neglect, is high [25, 26].

Exposure to different kinds of household dysfunctions has been reported to be one of the most serious risk factors for any type of abuse or neglect during childhood [37]. In this study, violence or a threat of violence towards students in terms of spanking, slapping, kicking or pushing by their parents or guardians was the most common form of abuse, experienced by more than three-quarters of participants. This finding is in contrary to those of other studies that reported the violent treatment of the mother as the most frequently reported form of household dysfunction.

Witnessing community violence is also identified as a cause of ACE [38]. In our study, 80 % (80%) of participants reported witnessing community violence at least once, most frequently by seeing or hearing someone being physically abused. This study shows that the chances of respondents being involved in physical fights or witnessing community violence increased with exposure to multiple forms of ACE, which reveals a trend similar to that of the Finkelhor ACE study [39].

Exposure to multiple forms of childhood maltreatment has been reported to have everlasting effects on mental health, which persist from adolescence to adulthood [10, 12, 25, 26]. In this study, multi-type maltreatment was positively associated with low self-esteem. This observation is in agreement with findings of other studies that reported similar findings [25, 26]. It is well known that childhood abuse adversely affects the personality characteristics of an individual in his/her childhood, adolescent and adult lives, and it lowers self-esteem [40]. It has been shown that having been exposed to physical abuse during childhood results in low self-esteem [41]. This finding is consistent with that of our study, in which physical abuse was negatively associated with self-esteem.

Similarly, in a study focusing on the long-term psychological results of childhood maltreatment, it was reported that emotional abuse (psychological abuse) lowers self-esteem levels and results in the development of depression [17–19]. Additionally, in a study by [42] that assessed forms multiple maltreatment, it was asserted that exposure to emotional abuse (psychological abuse) may cause low self-esteem.

Several studies have reported that exposure to sexual abuse during childhood consequently affects low self-esteem [43]. In a study that focused on the effects of female children 14–19 years of age being subjected to different types of sexual violence, it was reported that the self-esteem and depression levels of girls who have experienced rape is poorer than the self-esteem and depression levels of those were not exposed sexual violence, rape or an attempt of sexual coercion during that age range [44]. In our study, no statistically significant association was found between exposure to sexual abuse and self-esteem.

Childhood domestic violence exposure usually causes emotional trauma that is as severe as exposure to direct maltreatment. Studies have shown that witnessing domestic violence has a negative impact on a child’s well-being and health development, especially in relation to psychological aspects, such as low self-esteem [45]. In our study, household dysfunction was not significantly associated with low self-esteem.

One limitation of the study is that the study participants involved were secondary school students from randomly selected schools in five regions in Tanzania. Therefore, the results of this study cannot be generalized to a whole Tanzanian population. The data of multiple types of child maltreatment in our study are based on self-reports and recalls of participants over a period of many years. As such, biases cannot be excluded. Therefore, it is inevitable that interviewees will either underestimate or overestimate a situation when providing a self-report. Additionally, the cross-sectional design of this study limits the ability to infer causation in regard to the associations between multi-type child maltreatment and self-esteem.

## Conclusion

This study demonstrates that multi-type child maltreatment is unacceptably prevalent in Tanzania and negatively affects self-esteem among secondary school students (13–24 years old). This finding strongly suggests that studying individual types of maltreatment in isolation from other types may not capture a comprehensive picture of the problem.

We believe that this study adds to the recent body of literature on child maltreatment by holistically investigating multi-type child maltreatment, as opposed to examining a single type of maltreatment, as the majority of previous studies have investigated.

Furthermore, this study raises awareness of the prevalence of multi-type child maltreatment and incentivises policymakers to create policies that clearly stipulate that these forms of violence need to be avoided to improve the health of adolescents and adults.

### Limitation of the study

There are no available data to support the reliability and validity of the questionnaires in the (local) setting in which it was used.

## Abbreviations

ACE: Adverse Childhood Experiences
SPSS: Statistical Package for Social Sciences
USA: United States of America
WHO: World Health Organization
